# Changes in retail food environments around schools over 12 years and associations with overweight and obesity among children and adolescents in Flanders, Belgium

**DOI:** 10.1186/s12889-022-13970-8

**Published:** 2022-08-18

**Authors:** Vincent Smets, Stefanie Vandevijvere

**Affiliations:** Department of Public Health and Epidemiology, Sciensano (Scientific Institute of Public Health), J.Wytsmanstraat 14, 1050 Brussels, Belgium

**Keywords:** Food environments, Obesity, Convenience stores, Fast food restaurants, Children, Schools, Belgium

## Abstract

**Background:**

Children are susceptible to the food environment. This research assessed changes in retail food environments near schools in Flanders between 2008 and 2020 and associations with children’s and adolescents’ weight status.

**Methods:**

The food environment within a 500 m and 1000 m road network distance to all primary and secondary schools was mapped using spatial indicators. The commercial Locatus database, including addresses of all food retailers in Flanders, was used to calculate the density of different types of food retailers near the school perimeter, the percentage of schools with at least one food retailer of a certain type near the school perimeter and the shortest distance from the school entrance to the nearest food retailer of a certain type. A generalized linear model was used to explore associations between these indicators and the percentage of children and adolescents with overweight at the school level.

**Results:**

Food environments near schools in Flanders were found to be unhealthy in 2020, with a significant increase in fast food restaurants and convenience stores between 2008 and 2020. The density of fast food restaurants within a 1000 m walking distance from primary and secondary schools increased from 5.3 ±0.3 to 6.3 ±0.4 and from 10.2 ±0.7 to 12.7 ±0.9 respectively between 2008 and 2020, while the density of convenience stores increased from 3.2 ±0.3 to 3.8 ±0.4 and from 6.2 ±0.6 to 7.6 ±0.8 respectively.

Food environments near schools with a higher proportion of children from a poor socio-economic background were found unhealthier, regardless of the urbanization level. A significant positive association was found between the density of fast food restaurants as well as the density of convenience stores around primary schools and the percentage of children aged < 6 years and 6–12 years with overweight. A positive, not significant association was found between the density of fast food restaurants as well as the density of convenience stores around secondary schools and the percentage of adolescents, aged 13–14 and 15–18 years with overweight.

**Conclusion:**

Food environments around schools in Flanders became unhealthier over time and were associated with children’s weight status.

**Supplementary Information:**

The online version contains supplementary material available at 10.1186/s12889-022-13970-8.

## Background

Food environments have commonly been defined as ‘the physical, economic, political and socio-cultural contexts in which people engage with the food system to make their decisions about acquiring, preparing and consuming food’ [1, 2]. The concept of the food environment demonstrates how the choices that people make regarding the foods they eat are to a significant degree influenced by the context within which they are made. Most current food environments do not encourage healthy eating [3]. The obesity epidemic is at least partly a consequence of these unhealthy food environments [4]. In its series on obesity, The Lancet states that ‘Today’s food environments exploit people’s biological, physiological, social and economic vulnerabilities, making it easier for them to eat unhealthy foods’ [5].

Adequate, healthy nutrition is especially important for young children and adolescents, as their bodies and brains are still developing. The link between unhealthy diets during childhood and physical and mental health problems later in life has been clearly established [6, 7]. The prevalence of childhood obesity has increased dramatically worldwide since 1980 and is considered one of the most serious public health issues [8]. In Flanders, in 2018, it was observed that 16.2% of young people (2–17 years) were overweight and 4.6% of young people suffered from obesity [9].

About 19.4% of people in Flanders drank sugar sweetened beverages daily and 4.1% of people drank at least 1 L of those drinks daily in 2018 [10]. At all ages, the proportion of girls with overweight was higher than that of boys with overweight. In addition, children who were born or grew up in deprived areas had a less favorable weight status [11]. Around 20% of 4-year-olds in deprived areas in Flanders were overweight. Among non-privileged children, it was less than 1 in 10 at that age. From the age of 10 years onwards, at least 32% of children in deprived areas were overweight. This was twice as much as among children who did not grow up in poverty [11].

Besides their homes, children spend most of their time in and around their school. The healthiness of the food environment in and outside the school perimeter is therefor particularly important. In their recent systematic review, Matsuzaki et al. [12] found consistent positive associations between obesogenic food environments near schools and the body weight of children aged 6–18 years. Most studies they reviewed found positive associations between food environment indicators, such as the number of fast food restaurants or convenience stores near schools and children’s weight status. They also found limited evidence for negative associations between healthy food environments around schools and weight status among children [12].

In their meta-analysis of 100 studies about the food environment in and around schools, Pineda et al. [13] found that interventions addressing food environments inside schools, such as increasing the vegetable and fruit offerings in school canteens and the banning of vending machines that sell sugar sweetened beverages, have a positive effect on children’s weight status, but the authors note that the effect of these measures are partly nullified when the food environment around schools is obesogenic. The food environment around schools can influence the children’s meal choice in schools and therefor reduce the effectiveness of the measures addressing food environments in schools [13]. Fitzpatrick et al. [14] also note that the proximity of fast food restaurants and other unhealthy outlets near schools may lead to a greater exposure to unhealthy foods which can influence children’s eating habits.

The food environment inside schools may also influence the food environment around schools. Brunello et al. [15] investigated the effect of the European school fruit scheme which was implemented in schools by various EU member states by the end of 2008. They found that supermarkets within a 500 m radius around schools showed a significant decrease in the sales of junk food (6.3%) compared with supermarkets outside this radius. The effect was only observed for regular supermarkets and not for discount stores, leading the researchers to conclude that the campaign only had the intended effect in a subgroup of the population, namely the wealthier middle class who are less susceptible to overweight and obesity [15].

Studies have shown that children from families with low incomes generally consume less healthy diets and have higher BMI (Body Mass Index) z-scores [16, 17]. Several studies emphasize the stronger impact of unhealthy food environments on the weight status of children from poor families [12, 18]. Matsuzaki et al. [12] found in their systematic review a positive correlation between children’s weight status and the number of fast food restaurants in their school environment. This correlation was stronger for children from underprivileged families. Turbutt et al. [18] found in their review a strong correlation between the number of hot food takeaway restaurants and the socio-economic status of the area. Children who spend more time in deprived areas were found to eat more fast food and have a higher BMI [18].

When assessing the available evidence, it is clear that the food environment around schools has been much less studied than the food environment inside schools. Of the 100 studies that Pineda et al. [13] reviewed, only six looked at how the external food environment influenced the children’s weight status. A possible reason for this is that changing the food environment around schools is more complex and requires greater political will compared to changing the food environment inside schools. A better understanding and mapping of the food environment around schools and how it influences the children’s weight status is needed.

In Flanders, food environments inside schools have previously been studied by the institute Gezond Leven [19]. They found that education about healthy food is strongly embedded in the curriculum of all primary and secondary schools and that most schools implemented some health promoting measures, such as reducing their offerings of sugar sweetened beverages [19]. There is however still a lot of progress to be made. For example only 37% of secondary schools did not offer unhealthy snacks and only 36% of secondary schools phased out sugar sweetened beverages [20].

This study is the first one to map the food environment around primary and secondary schools in Flanders and evaluate trends over time and associations with children’s and adolescents’ weight status. The objectives of this study were I) To assess retail food environments in Flanders near schools (situation 2020, pre-covid19) II) To assess if and how retail food environments near schools in Flanders changed between 2008 and 2020 and III) To determine if there are associations between retail food environments in Flanders near schools, the socio-economic status of the schools’ children and the children’s weight status.

## Methods

### Study area

Belgium consists of three major regions: Flanders, Brussels and Wallonia. Flanders is an area in the northern part of Belgium. The region inhabits 6.629.143 people (57.5% of Belgian population), of which 1.123.000 are of school going age (2.5 to 17 years) [21]. The region is strongly urbanized with a high population density (383.9 inhabitants/km^2^). This study comprised the entire territory of Flanders, including all schools and food stores and services in Flanders.

### Data sources

#### Food outlets

The geographical coordinates of all food retailers (stores and services) in Flanders were obtained from the commercial Locatus database (www.locatus.com). It covers the entire territory of Flanders and includes information such as the name and address of the retailer, the type of outlet and the size of the retail space. Since 2008, Locatus systematically performs regular field audits in Belgium to map the locations, sizes and types of retailers for commercial purposes. The frequency of field audits varies from once a year—in shopping areas—to once every 2 or 3 years in locations outside shopping areas.

Locatus has its own field service staff, who visit, inventorize and check all points of sale in the Benelux on a yearly basis. For this study the databases from the years 2008, 2013 and 2020 were acquired. The original database included 33 types of retailers whose primary purpose is to sell food, which was too detailed for the purposes of this study. Based on a consultation with an expert committee in Flanders consisting of 15 dieticians, food scientists and food policy advisors, a reclassification was performed into nine different classes of food outlets for the purposes of this study. The nine resulting classes of food retailers were: supermarkets, confectionary stores, convenience stores, fast food/takeaway/delivery outlets, shops that primarily sell animal products, full service restaurants, greengrocers, bakeries and other shops. The complete reclassification can be consulted in Additional file 1.

#### Road network and urbanicity

The road network of Flanders was sourced from the ‘Grootschalig Referentie Bestand Vlaanderen (GRB)’, which is a freely available data source, managed by the Flemish government, that continually maps and updates spatial entities such as roads, buildings and waterways [22].

The Degree of urbanization (DEGURBA) dataset, provided by Eurostat, was used to divide Flanders in regions that are ‘essentially urban’, ‘intermediate’ and ‘essentially rural’ [23].

#### Schools

The school data was acquired from the Department for Education Flanders and included the schools’ unique ID, name, type and geographical coordinates. The food environment was analyzed for both primary (*n* = 3404, children aged 2.5–12 years) and secondary (n = 1195, children aged 13–18 years) schools.

An anonymized dataset with aggregated socio-economic status (SES) indicators for the students of every school in Flanders was provided by the Flemish government. The SES indicators available included the proportion of pupils/students for which the level of education of the mother was low (i.e. defined as not completing a secondary school education) and the proportion of pupils/students for which the home language was not Dutch. Previous research has demonstrated that these indicators are strong predictors for children’s mental wellbeing, cognitive function and adiposity [24–26]. The schools were divided into terciles (‘low’, ‘medium’, ‘high’) for both SES indicators. The upper tercile (‘high’ score) means that the proportion of the schools’ children with a low educated mother or for whom the home language is not Dutch is high.

#### Weight status

A dataset including the percentage of children with overweight for each school and for the years 2011–2016 was provided to the researchers by the Flemish government agency ‘Agentschap Zorg en Gezondheid (AZG)’.

These data were stratified by sex and age group (< 6 years, 6-12 years, 13–14 years and 14–18 years) (See Additional file 2). Due to privacy reasons it was not possible to obtain the data on weight status, including BMI z-scores, from the individual children, only aggregated data (% of children with overweight) at the school level were accessible for research. In the analyses, therefore, the percentage of children with overweight at the school level was used.

The height and weight of all children were measured on a yearly basis when they visit the centers for pupils support (CLBs). These measures are obligatory as determined by a government decree, so parents or children cannot opt out unless they are sick on the day of the measurements. Height was measured barefoot, in light clothing (no jumper, shirt or jacket) with a SECA 213 mobile stadiometer. Weight was measured in underwear at a precision of at least 100 g with a SECA877 or Seca Quadra 808 scale. The percentage of children with overweight for each of the schools was calculated by AZG through comparing the BMI of the individual children with the IOTF thresholds for overweight and obesity [27] and those of the WHO (for low BMI for age).

### Selection of indicators of the food environment

The first indicator that was calculated was the absolute density (number) of each food retail type within a 500 m and 1000 m road network distance from the main entrance of the school. The 500 m and 1000 m road network distances were chosen based on common walking distances that most children can do fairly easily. At an average walking speed of 5 km/h, a person would need approximately 6 or 12 min to walk 500 m or 1000 m respectively. Other studies in the same context, such as Cant et al. [28], assumed the same common walking distances.

The second indicator that was computed was the percentage of schools that had at least one food retailer of a certain type within a walking distance of 500 m or 1000 m from the entrance of the school.

The final indicator that was calculated was the shortest distance from each school entrance to the nearest fast food outlet, supermarket and convenience- or confectionary store. These types of food retailers were identified to be the most probable types of food shops that children would visit during lunchbreak or outside of school hours.

### Geographical analyses

The analyses were performed in QGIS 3.16.5 and PostGIS 3.1. The service area algorithm in QGIS created a road distance network of 500 m and 1000 m around the entrance of every primary and secondary school. The absolute density of each retail type and the percentage of schools that had at least one food retailer of a certain type in their food environment were calculated based on these road distance networks. The Dijkstra algorithm, implemented in the pgRouting package (PostGIS), was used to calculate the shortest network distances needed for the third indicator. A custom SQL query was written to automate the process and perform the analyses for all schools simultaneously.

### Statistical analyses

The statistical analyses were performed with SAS9.4. Associations between the density of fast food, takeaway, delivery outlets around schools, and the percentage of children with overweight was determined through a generalized linear model adjusted for the level of urbanization (DEGURBA), socio-economic status of children at school level (proportion of children for whom education level of the mother is low) and sex. The same analysis was repeated for convenience stores instead of fast food, takeaway and delivery outlets. All analyses were stratified by age group (< 6 years, 6–12 years, 13–14 years and 15–18 years) and by buffer size (500 m/1000 m road network distance from the school). A lag time of zero, one and two years was considered for assessing the association between exposure to fast food, takeaway, delivery outlets as well as convenience stores and the percentage of children with overweight.

Children’s data on weight status from the school years 2010–11, 2013–14, 2014–15 and 2015–16 were used and linked with Locatus data from the years 2008 and 2013.

In view of the many analyses conducted, a Bonferroni correction was applied, where a *p*-value of < 0.0006 (0.05/88) was considered statistically significant.

## Results

### Trends in retail food environments around schools in Flanders

The mean absolute density of different food retail types within a 500 m and 1000 m walking distance buffer from the school entrance can be found in Table 1 (primary schools) and Table 2 (secondary schools). The absolute density of traditional stores around schools such as bakeries, local shops selling animal products and greengrocers significantly decreased between 2008 and 2020. In the case of greengrocers, their numbers approximately halved from 0.29 stores to 0.14 stores on average within a 500 m walking distance buffer from primary schools (Tables 1 and 2).

**Table 1:** Mean absolute density (i.e. number of outlets) of different types of food retailers within 500 m and 1000 m road network distance from the main entrance of primary schools (*N* = 3404) in Flanders (year = 2008 & 2020) – Significant results comparing 2008 & 2020 indicated in bold

*buffer*	*food outlet type*	*2008*				*2020*			
		*mean*	*Lower 95%CI*	*Upper 95%CI*	*max*	*mean*	*Lower 95%CI*	*Upper 95%CI*	*max*
*500 m*	*Shops selling animal products*	***1.22***	***1.16***	***1.28***	***18***	***0.84***	***0.79***	***0.89***	***20***
*500 m*	*Bakeries*	***1.50***	***1.44***	***1.56***	***15***	***1.17***	***1.12***	***1.22***	***20***
*500 m*	*Confectionary Stores*	*0.43*	*0.38*	*0.48*	*44*	*0.47*	*0.41*	*0.53*	*61*
*500 m*	*Convenience Stores*	***1.02***	***0.93***	***1.11***	***44***	***1.25***	***1.13***	***1.37***	***65***
*500 m*	*Fast food/takeaway/delivery outlets*	***1.86***	***1.77***	***1.95***	***34***	***2.19***	***2.06***	***2.31***	***42***
*500 m*	*Full Service Restaurants*	*3.54*	*3.22*	*3.87*	*166*	*4.24*	*3.87*	*4.62*	*191*
*500 m*	*Greengrocers*	***0.29***	***0.27***	***0.31***	***4***	***0.14***	***0.12***	***0.15***	***4***
*500 m*	*Other Shops*	***0.14***	***0.13***	***0.16***	***3***	***0.07***	***0.06***	***0.08***	***2***
*500 m*	*Supermarkets*	*0.60*	*0.58*	*0.63*	*7*	*0.65*	*0.61*	*0.68*	*10*
*1000 m*	*Shops selling animal products*	***3.25***	***3.10***	***3.40***	***40***	***2.29***	***2.17***	***2.40***	***35***
*1000 m*	*Bakeries*	***3.76***	***3.61***	***3.91***	***34***	***2.93***	***2.80***	***3.06***	***38***
*1000 m*	*Confectionary Stores*	*1.28*	*1.16*	*1.40*	*52*	*1.46*	*1.31*	*1.62*	*70*
*1000 m*	*Convenience Stores*	*3.19*	*2.90*	*3.48*	*106*	*3.83*	*3.47*	*4.19*	*143*
*1000 m*	*Fast food/takeaway/delivery outlets*	***5.28***	***5.01***	***5.56***	***91***	***6.34***	***5.96***	***6.73***	***111***
*1000 m*	*Full Service Restaurants*	*11.63*	*10.60*	*12.67*	*387*	*13.80*	*12.58*	*15.03*	*420*
*1000 m*	*Greengrocers*	***0.80***	***0.75***	***0.84***	***9***	***0.41***	***0.38***	***0.44***	***10***
*1000 m*	*Other Shops*	***0.42***	***0.39***	***0.45***	***9***	***0.22***	***0.20***	***0.23***	***6***
*1000 m*	*Supermarkets*	***1.73***	***1.66***	***1.80***	***16***	***2.09***	***1.98***	***2.19***	***28***

**Table 2:** Mean absolute density (i.e. number of outlets) of different types of food retailers within 500 m and 1000 m road network distance from the main entrance of secondary schools (*N* = 1195) in Flanders (year = 2008 & 2020) – Significant results comparing 2008 & 2020 indicated in bold

*buffer*	*food outlet type*	*2008*				*2020*			
		*mean*	*Lower 95%CI*	*Upper 95%CI*	*max*	*mean*	*Lower 95%CI*	*Upper 95%CI*	*max*
*500 m*	*Shops selling animal products*	***1.68***	***1.56***	***1.81***	***17***	***1.18***	***1.08***	***1.27***	***14***
*500 m*	*Bakeries*	***1.92***	***1.80***	***2.04***	***13***	***1.48***	***1.38***	***1.59***	***11***
*500 m*	*Confectionary Stores*	*0.99*	*0.85*	*1.14*	*44*	*1.07*	*0.90*	*1.25*	*61*
*500 m*	*Convenience Stores*	***1.62***	***1.44***	***1.80***	***45***	***2.03***	***1.82***	***2.25***	***55***
*500 m*	*Fast food/ takeaway/delivery outlets*	***3.05***	***2.83***	***3.27***	***39***	***3.68***	***3.40***	***3.97***	***41***
*500 m*	*Full Service Restaurants*	*8.03*	*7.17*	*8.89*	*152*	*9.67*	*8.68*	*10.66*	*172*
*500 m*	*Greengrocers*	***0.41***	***0.37***	***0.45***	***4***	***0.23***	***0.20***	***0.26***	***4***
*500 m*	*Other Shops*	***0.29***	***0.26***	***0.32***	***4***	***0.13***	***0.11***	***0.15***	***2***
*500 m*	*Supermarkets*	***0.76***	***0.71***	***0.82***	***6***	***0.97***	***0.89***	***1.04***	***9***
*1000 m*	*Shops selling animal products*	***5.69***	***5.36***	***6.01***	***39***	***4.04***	***3.78***	***4.29***	***33***
*1000 m*	*Bakeries*	***6.37***	***6.04***	***6.70***	***36***	***4.90***	***4.62***	***5.17***	***37***
*1000 m*	*Confectionary Stores*	*3.23*	*2.89*	*3.57*	*53*	*3.77*	*3.31*	*4.24*	*70*
*1000 m*	*Convenience Stores*	***6.15***	***5.52***	***6.79***	***99***	***7.61***	***6.81***	***8.42***	***138***
*1000 m*	*Fast food/takeaway/delivery outlets*	***10.21***	***9.56***	***10.86***	***88***	***12.68***	***11.76***	***13.60***	***113***
*1000 m*	*Full Service Restaurants*	*28.69*	*25.83*	*31.56*	*363*	*34.12*	*30.80*	*37.44*	*409*
*1000 m*	*Greengrocers*	***1.37***	***1.27***	***1.46***	***9***	***0.70***	***0.63***	***0.77***	***10***
*1000 m*	*Other Shops*	***0.96***	***0.89***	***1.04***	***9***	***0.45***	***0.41***	***0.49***	***5***
*1000 m*	*Supermarkets*	***2.69***	***2.55***	***2.83***	***16***	***3.61***	***3.39***	***3.83***	***30***

On the other hand, food retail types such as fast food, takeaway and delivery outlets, convenience stores and supermarkets saw significant increases between 2008 and 2020. For example, the mean density of fast food, takeaway and delivery outlets within a 500 m walking distance from the school entrance of primary schools increased significantly from 1.86 stores to 2.19 stores between 2008 and 2020. Full service restaurants were most ubiquitous around primary and secondary schools, although their increase between 2008 and 2020 was not significant. They were followed by fast food, takeaway and delivery outlets, bakeries and local stores selling animal products (Tables 1 and 2). When comparing the densities of retail types around primary and secondary schools (Table 2) higher densities were observed for secondary schools.

Similar results were found for the indicator on the percentage of schools with at least one outlet of a certain retail type within a walking distance of 500 m or 1000 m from the entrance of the school (Table 3). A significant decrease was observed for the percentage of schools with at least one traditional food outlet (bakeries, shops selling animal products and greengrocers) while the percentage of schools with at least one other food outlet, such as full service restaurants and convenience stores increased between 2008 and 2020. The percentage of primary and secondary schools with at least one fast food, delivery or takeaway outlet within a walking distance of 1000 m was 82.9% and 90.9% in 2020 respectively (Table 3).

**Table 3:** Percentage of primary/secondary schools with at least one food outlet from the different retail types within 500 m and 1000 m road network distance from the main entrance of the schools (year = 2008 & 2020)

*Food outlet type*	*School type*	*500 m walking distance from school entrance*		*1000 m walking distance from school entrance*	
		*2008*	*2020*	*2008*	*2020*
*Full Service Restaurants*	*primary*	*58.3*	*62.7*	*80.1*	*83.3*
	*secondary*	*70.5*	*73.7*	*91.1*	*92.6*
*Fast Food/Takeaway/Delivery*	*primary*	*61.5*	*64.0*	*81.1*	*82.9*
	*secondary*	*69.0*	*70.8*	*89.7*	*90.9*
*Confectionary Stores*	*primary*	*20.3*	*21.7*	*40.0*	*40.8*
	*secondary*	*36.4*	*36.5*	*66.6*	*66.6*
*Bakeries*	*primary*	*66.0*	*60.6*	*83.6*	*79.4*
	*secondary*	*66.1*	*61.3*	*88.5*	*86.0*
*Supermarkets*	*primary*	*43.0*	*40.9*	*70.0*	*68.0*
	*secondary*	*50.5*	*53.0*	*82.3*	*85.3*
*Shops selling animal products*	*primary*	*56.5*	*45.8*	*76.6*	*69.0*
	*secondary*	*61.3*	*52.6*	*84.9*	*81.7*
*Convenience Stores*	*primary*	*40.3*	*43.6*	*59.1*	*62.0*
	*secondary*	*49.9*	*56.3*	*75.6*	*80.3*
*Greengrocers*	*primary*	*22.8*	*11.6*	*43.6*	*25.9*
	*secondary*	*30.9*	*18.0*	*61.3*	*40.1*
*Other Shops*	*primary*	*11.9*	*7.1*	*26.6*	*17.8*
	*secondary*	*22.1*	*12.0*	*51.5*	*33.6*

With regards to the shortest median distance from the school entrance to the nearest retail outlet of a certain type, the differences between 2008 and 2020 were not significant. This means that the median distance that children needed to walk to reach one of these outlets did not change since 2008. Shortest median distances were between 300 and 500 m for most outlet types, meaning that children on average needed to walk between 4–6 min from the school entrance to reach the nearest outlet.

For instance, the median walking distance from primary and secondary schools to the nearest fast food outlet was 369.2 m (IQR 202 m-728.4 m) and 325.3 m (IQR 172.7 m-574.7 m) in 2020 respectively, while the median distance to the nearest convenience or confectionary store was 525.9 m (IQR 263.1 m- 1464.0 m) and 379.8 m (IQR 208.5 m-749.3 m) respectively.

The median distance to the nearest supermarket was 640.3 m (IQR 353.7 m-1432.4 m) for primary schools and 501.3 m (IQR 321.3 m-747.1 m) for secondary schools. The distances were lower for secondary compared to primary schools, meaning that older children had easier access to unhealthy foods. The table with the full results and interquartile ranges can be found in Additional file 3.

### Associations between food environment indicators, socio-economic status and the level of urbanization

**Fig. 1:**
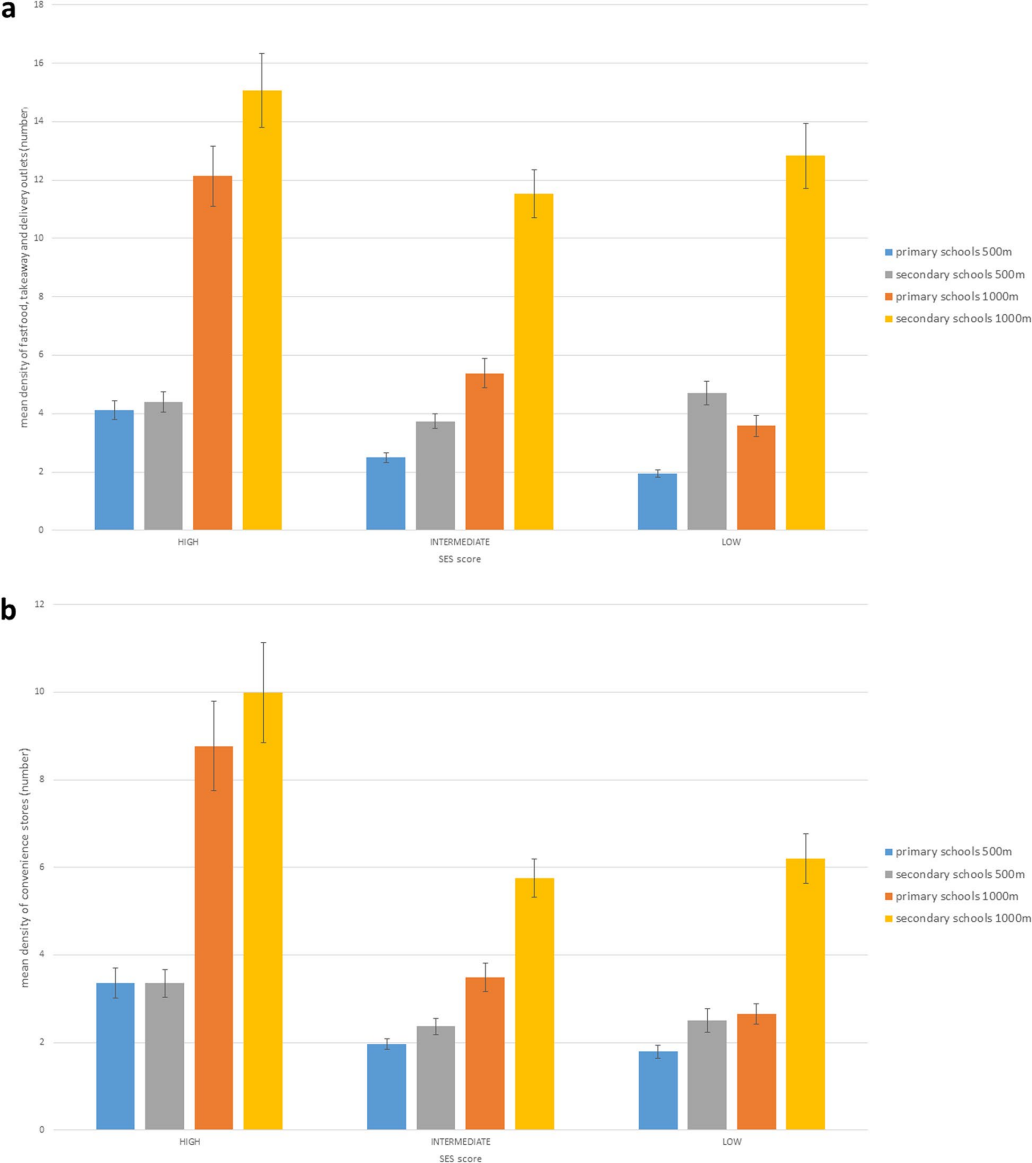
The mean absolute density (95% CI) of **a**) fast food/takeaway and delivery outlets and **b**) convenience stores within a 500 m and 1000 m walking distance from the school entrance of primary schools and secondary schools (year 2020) according to a high, medium or low percentage of students with a low educated mother

Schools that have a high % of pupils who have a low educated mother or whose home language is not Dutch, have unhealthier food environments with a higher density of fast food, takeaway and delivery outlets and convenience stores compared to schools with a medium or low % of pupils from lower socioeconomic backgrounds (Fig. 1). This gradient was most pronounced for primary schools. For example, a primary school in Flanders that has a high % of pupils with a lower educated mother had on average 12.1 ±1.0 fast food, takeaway or delivery outlets and on average 8.8 ±1.0 convenience stores within a 1000 m walking distance, while a primary school that has a low % of pupils with a lower educated mother had on average only 3.6 ±0.4 fast food, takeaway or delivery outlets and on average 2.6 + -0.24 convenience stores nearby. This gradient remained when the results were corrected for the level of urbanization, although the density of stores was lower outside urban centers (Additional file 4). The results for the % of pupils whose home language is not Dutch were similar and can be found in Additional file 4.

**Fig. 2:**
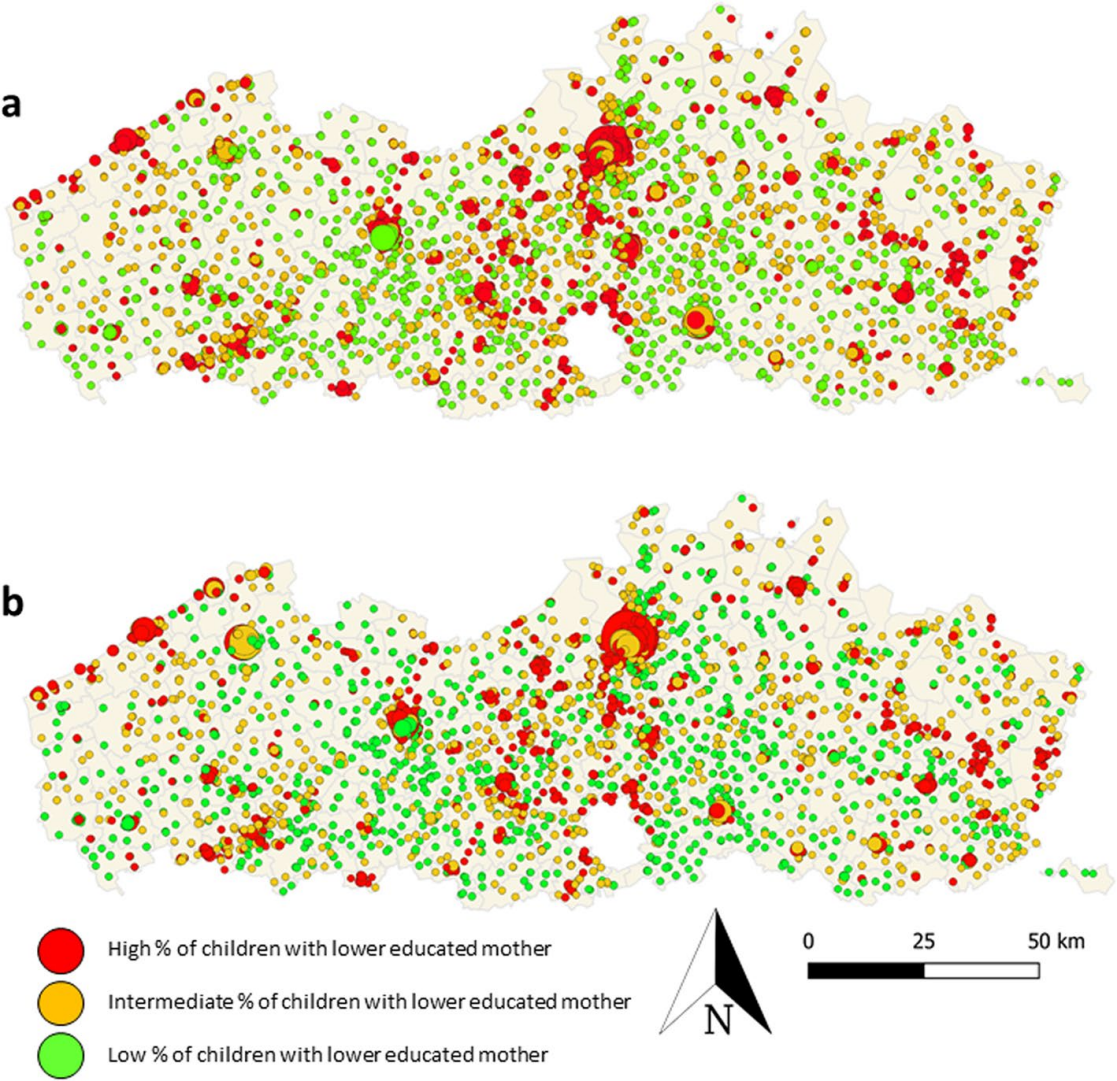
Location of primary schools in Flanders. The size of the circle varies depending on **a**) the density of fast food/takeaway and delivery outlets and **b**) convenience stores within a 1000 m walking distance from the school entrance. The color of the circle shows if the school as a high, medium or low percentage of students with a low educated mother (year 2020)

The following map shows the geographical relationship between the density of fast food, takeaway and delivery outlets and convenience stores within a 1000 m walking distance from primary schools and the % of pupils that have a low educated mother (Fig. 2).

The schools with a high density of fast food, takeaway and delivery outlets and convenience stores were predominantly located in urban city centers such as Antwerp, Gent and Leuven. Besides a few exceptions in Gent, all these schools were colored orange or red, meaning that they have a medium or high % of children with a low educated mother.

### Associations between food environments near schools and children’s weight status

The percentage of children with overweight (Additional file 2) was provided by AZG by school for each of the school years 2010–2011 until 2015–2016 and by age group (younger than 6 years, 6–12 years, 13–14 years and 15–18 years). The percentage of children with overweight at the school level was used as the dependent variable in the generalized linear models.

The results of the models that link the density of fast food, takeaway and delivery outlets around schools with the percentage of the schools’ children with overweight can be found in Table 4.

**Table 4:** Associations between the density of fast food/takeaway/delivery outlets around schools and the percentage of the schools’ children with overweight, by age group and buffer size (500 m/1000 m), adjusted for sex, level of urbanicity of municipality and % of pupils with a low educated mother

school year	Locatus year	outlet	buffer	indicator weight status	Age group	Crude		Adjusted	
						Coefficient/SE	p	Coefficient/SE	p
2010–11	2008	fast food	500 m	% overweight	Younger than 6 years	0.282 /0.032	< 0.001	0.118 / 0.032	0.001
2010–11	2008	fast food	500 m	% overweight	6–12 years	0.190 / 0.028	< 0.001	0.084 /0.028	0.003
2010–11	2008	fast food	500 m	% overweight	13–14 years	-0.026 / 0.044	0.804	-0.023 / 0.043	0.598
2010–11	2008	fast food	500 m	% overweight	15–18 years	0.049 / 0.067	0.466	0.102 / 0.070	0.148
2010–11	2008	fast food	1000 m	% overweight	Younger than 6 years	0.107 / 0.010	< 0.001	0.044 / 0.011	< 0.001
2010–11	2008	fast food	1000 m	% overweight	6–12 years	0.099 / 0.009	< 0.001	0.048 /0.010	< 0.001
2010–11	2008	fast food	1000 m	% overweight	13–14 years	0.028 / 0.015	0.061	0.004 /0.016	0.814
2010–11	2008	fast food	1000 m	% overweight	15–18 years	0.030 / 0.023	0.190	0.044 / 0.025	0.087
2015–16	2013	fast food	500 m	% overweight	Younger than 6 years	0.246 / 0.029	< 0.001	0.076 / 0.028	< 0.001
2015–16	2013	fast food	500 m	% overweight	6–12 years	0.268 / 0.026	< 0.001	0.097 / 0.025	< 0.001
2015–16	2013	fast food	500 m	% overweight	13–14 years	0.035 /0.046	0.451	0.029/ 0.044	0.504
2015–16	2013	fast food	500 m	% overweight	15–18 years	-0.011 / 0.068	0.872	-0.045 / 0.069	0.512
2015–16	2013	fast food	1000 m	% overweight	Younger than 6 years	0.111 / 0.009	< 0.001	0.046 / 0.010	< 0.001
2015–16	2013	fast food	1000 m	% overweight	6–12 years	0.117 / 0.008	< 0.001	0.043 / 0.008	< 0.001
2015–16	2013	fast food	1000 m	% overweight	13–14 years	0.045 / 0.015	0.002	0.010 / 0.015	0.521
2015–16	2013	fast food	1000 m	% overweight	15–18 years	0.009 / 0.021	0.681	-0.013 / 0.022	0.574
2013–14	2013	fast food	500 m	% overweight	Younger than 6 years	0.261 / 0.29	< 0.001	0.068 / 0.029	0.018
2013–14	2013	fast food	500 m	% overweight	6–12 years	0.249 / 0.026	< 0.001	0.118 / 0.024	< 0.001
2013–14	2013	fast food	500 m	% overweight	13–14 years	0.041 / 0.045	0.371	0.089 / 0.044	0.045
2013–14	2013	fast food	500 m	% overweight	15–18 years	0.018 / 0.066	0.786	0.065 / 0.067	0.331
2013–14	2013	fast food	1000 m	% overweight	Younger than 6 years	0.107 / 0.009	< 0.001	0.031 / 0.010	0.002
2013–14	2013	fast food	1000 m	% overweight	6–12 years	0.102 / 0.008	< 0.001	0.035 / 0.008	< 0.001
2013–14	2013	fast food	1000 m	% overweight	13–14 years	0.036 / 0.014	0.013	0.026 / 0.015	0.089
2013–14	2013	fast food	1000 m	% overweight	15–18 years	0.016 / 0.020	0.421	0.022 / 0.022	0.308
2014–15	2013	fast food	500 m	% overweight	Younger than 6 years	0.273 / 0.028	< 0.001	0.100 / 0.033	0.003
2014–15	2013	fast food	500 m	% overweight	6–12 years	0.197 / 0.023	< 0.001	0.080 / 0.026	0.002
2014–15	2013	fast food	500 m	% overweight	13–14 years	-0.014 / 0.044	0.749	0.050 / 0.048	0.305
2014–15	2013	fast food	500 m	% overweight	15–18 years	-0.121 / 0.064	0.059	-0.111 / 0.075	0.138
2014–15	2013	fast food	1000 m	% overweight	Younger than 6 years	0.114 / 0.009	< 0.001	0.053 / 0.010	< 0.001
2014–15	2013	fast food	1000 m	% overweight	6–12 years	0.101 / 0.008	< 0.001	0.046 / 0.008	< 0.001
2014–15	2013	fast food	1000 m	% overweight	13–14 years	0.025 / 0.014	0.082	0.030 / 0.016	0.054
2014–15	2013	fast food	1000 m	% overweight	15–18 years	0.006 / 0.021	0.779	-0.006 / 0.024	0.806

All models showed a positive association between the density of fast food, takeaway and delivery outlets and the percentage of children < 6 years and 6–12 years with overweight (for the majority of models the association is significant after applying the Bonferroni correction *p* < 0.0006). This was the case for both buffers (500 m/1000 m) and the three lag periods (zero, one or two years). The results were similar for convenience stores (see Additional file 5).

For 13–14 year old adolescents, when corrected for multiple testing, there were no significant associations between the density of fast food, takeaway and delivery outlets around schools and the percentage of adolescents with overweight. There was only a significant association between to the density of convenience stores and the percentage of adolescents with overweight for the one-year lag time (Table 4).

For 15–18 year old adolescents, when corrected for multiple testing, there were no associations between the density of fast food, takeaway and delivery outlets around schools and the percentage of adolescents with overweight, regardless of the lag time taken into account (Table 4). The results for convenience stores were similar and can be found in Additional file 5.

## Discussion

This study showed that food environments around schools in Flanders were generally unhealthy with children having an increasingly higher exposure to unhealthy food retailers such as convenience stores and fast food, takeaway and delivery outlets. In addition, the number of traditional stores such as bakeries, greengrocers and stores that sell animal products around schools decreased over a 12-year period. These outlets were replaced by full service restaurants, convenience stores, fast food, takeaway and delivery outlets and confectionary stores which significantly increased over the same time period. These changes probably reflect a broader change in the food landscape in Flanders and are similar to results found in the Netherlands [29].

The differences in indicator values between primary and secondary schools are likely due to the fact that primary schools are often located in semi-urban areas while most secondary schools are located in denser, urban areas. Hence the density of food retailers around secondary schools was higher and walking distances were shorter than for primary schools.

Food environments near schools with a higher proportion of pupils from lower socio-economic backgrounds were found to be unhealthier. This gradient was most pronounced for primary schools and remained after correcting for the level of urbanization.

Students from these schools often come from impoverished families that lack knowledge about a healthy nutritious diet and therefor are extra vulnerable to succumb to the allure of unhealthy food environments [12, 18]. The reason that food environments near these schools were unhealthier is likely due to supply and demand effects. Because children from impoverished families often eat more unhealthy at home, they are also more likely to eat less healthy outside their home environment [30]. Food retailers that offer unhealthy food will be drawn more to the schools where these children go to [12], giving them plenty of opportunities to continue to consume the diet they are used to at home, thus creating a vicious cycle.

A significant positive association was found between the density of fast food, takeaway, delivery outlets, as well as density of convenience stores near schools and the % of children with overweight for children < 6 years and 6–12 years but not for adolescents 13–14 years and 15–18 years. Children < 6 years and 6–12 years generally commute to their school under parental supervision and cannot leave the school during lunch break. On a first glance they interact less with the food environment near their school than older children who are often allowed to leave the school during lunch break and are more independent and free to determine their own food choices. Research however has shown that solely being exposed to an unhealthy food environment can lead to a more unhealthy diet [31, 32].

Young children are very susceptible to marketing and merely passing through an unhealthy food environment twice a day can lead them to craving more sugary snacks and/or fatty foods, influencing their parents’ purchasing behavior. Older children (13–14 years and 15–18 years) on the other hand, are much more mobile and are not necessarily restricted to the food environment near their school, but interact with many more different food environments. Around this age, they often experience a growth spurt meaning that an unhealthy diet won’t necessarily show in their weight status right away but might create dietary habits that can lead to excess weight later in life.

Previous research has shown that a high BMI and an increased prevalence of cardio metabolic risk factors such as high levels of LDL cholesterol or elevated blood pressure are not always correlated [33]. Therefor adolescents with a normal BMI can possibly still have high levels of LDL cholesterol or an increased blood pressure. This research is unable to determine this effect because these variables are not available.

Possible measures to improve the food environment around schools that local municipalities can take are to limit the number of unhealthy outlets or forbid the establishment of new unhealthy outlets near a school. It is crucial that the Flemish government revises local planning and zoning policies to give local policymakers the appropriate legal instruments.

Examples from other countries are ubiquitous: For example since 2010, several districts in London have established exclusion zones around target locations such as local centers, parks and schools, banning hot food takeaways in these zones completely [34]. In total 165 (50.5%) of local government areas in England have a specific policy targeting fast food outlets, of which 56 (34.1%) are health focused [35]. In Korea, Green Food Zones were created in 2009 within a 200 m perimeter around schools to construct a safe and sanitary food environment [36]. The Green Food Zones are managed by supervisors and the sale of low quality food for children was banned. In 2008, Los Angeles implemented a one year moratorium on opening or expanding fast food outlets in the South region [37].

Local authorities can also oblige unhealthy outlets to offer a minimum amount of healthy options at a lower price than the unhealthy ones. Another option is to limit opening hours of unhealthy food outlets before and after school hours. Governmental programs to change the food environment inside schools are likely to also have an effect on the outside school environment [15]. Any governmental attempt to change the external food environment is likely to meet with some resistance, both from shop owners and the general public. It is therefore important that government, when implementing these measures, informs the public behind the reason of the measure.

This research provides policy makers in Flanders with important data about the food environment near schools, which can be used as a foundation to create policies aimed at improving the food environment. Recommendations for further research include measuring additional health indicators such as the children’s and adolescents' cholesterol level, triglycerides, blood pressure, etc. Another suggestion for future studies is to explore the link between the food environment outside schools and the food environment inside schools and the combined impact they have on children’s weight status.

This study has some key strengths and limitations. This is the first comprehensive survey on retail food environments around schools in Flanders, using the largest assembled food outlet list. The Locatus data has previously been found accurate in a Dutch validation study [38]. In addition, measured weight and height data from a large sample of children and adolescents in Flanders was used. It is important to note that these data were anonymized, aggregated and stratified by sex and age group at the school level by the Flemish government agency ‘Agentschap Zorg en Gezondheid’. Due to privacy reasons, data on weight status of individual children were not available for research. Hence, the nature of the data did not allow for within-school cluster analysis using the individual’s children BMI z-score, neither did it allow for longitudinal statistical analysis that would account for repeated measurements of adiposity on the same children. Although 500 m and 1000 m are common walking distances, the definition of neighborhoods in spatial studies, such as in this study, is quite arbitrary. The study setup did not allow to map individual children’s journeys from home to school and back through the food environment, neither did it account for other individual changes in risk factors for obesity such as exercise, sleep and stress.

## Conclusion

The food environment near schools in Flanders was characterized comprehensively for the first time using three spatial indicators and was found to have become more unhealthy between 2008 and 2020, with children having easy access to unhealthy food outlets such as fast food restaurants and convenience stores. This result is exacerbated for schools with a high % of children with a low educated mother or whose home language is not Dutch, making these children extra susceptible for the detrimental health consequences of an unhealthy food environment. Our findings show that there is a significant positive association between the density of fast food restaurants, as well as convenience stores and the % of children with overweight for children < 6 years and 6- 12 years. For adolescents aged 13–14 years and 15–18 years no significant associations were found. Policymakers are advised to use these results to formulate policies to improve the food environment near schools and protect children’s health.

## Supplementary Information

**Additional file 1:** **Table S1.** The original food retail categories from the Locatus database and their reclassification for the purposes of the study.

**Additional file 2:** **Table S2.** The average percentage of Flemish children with overweight stratified by age group and sex and school year.

**Additional file 3:** **Table S3.** Median (IQR) shortest distance (in meters) from primary/secondary schools to the closest convenience store, fast food/takeaway/delivery outlet and supermarket (years 2008 & 2020).

**Additional file 4:** **Figure S1.** Mean absolute density of fast food, takeaway and delivery outlets within 500m and 1000m road network distance from the entrance of primary schools in Flanders (year=2020) according to low, medium and high percentage of pupils with home language not being Dutch by level of urbanization of the municipality where the school is located. **Figure S2.** Mean absolute density (with 95% CI) of fast food, takeaway and delivery outlets within 500m and 1000m road network distance from the entrance of primary/secondary schools in Flanders (year=2020) according to low, medium and high percentage of pupils whose home language is not Dutch. **Figure S3.** Mean absolute density (with 95% CI) of convenience stores within 500m and 1000m road network distance from the entrance of primary/secondary schools in Flanders (year=2020) according to low, medium and high percentage of pupils whose home language is not Dutch. **Figure S4.** Mean absolute density of fast food, takeaway and delivery outlets within 500m and 1000m road network distance from the entrance of primary schools in Flanders (year=2020) according to low, medium and high percentage of pupils with a low educated mother by level of urbanization of the municipality where the school is located.

**Additional file 5:** **Table S4.** Associations between the density of convenience stores around schools and the percentage of the schools’ children with overweight, by age group and buffer (500m/1000m), adjusted for sex, level of urbanicity of municipality and % of pupils with a low educated mother.

## Data Availability

The datasets used and/or analysed during the current study are available from the corresponding author on reasonable request.

## Abbreviations

BMI: Body Mass Index
SES: Socio-Economic Status indicators
