# Supplementary material for: Changes in retail food environments around schools over 12 years and associations with overweight and obesity among children and adolescents in Flanders, Belgium
Source: BMC Public Health. 2022 Aug 18;22:1570. doi: 10.1186/s12889-022-13970-8 (PMC9387020; doi:10.1186/s12889-022-13970-8)
Supplement: Supplementary file 1 — Additional file 1: Table S1. The original food retail categories from the Locatus database and their reclassification for the purposes of the study. [file 12889_2022_13970_MOESM1_ESM.docx]

**Supporting information**

**Additional file 1: Reclassification of the Locatus food retail outlet categories**

*Table S1: The original food retail categories from the Locatus database and their reclassification for the purposes of the study*

| Reclassification | Locatus classification | Locatus definition |
| --- | --- | --- |
| Fastfood /takeaway/delivery outlets | 59.210.171-Fastfood | Meal provider where service is not at the table, without fixed cutlery and where the usually fried products are ready for consumption within minutes of ordering (excludes sandwich stores) |
|  | 59.210.215-Grillroom/ Shoarma | Sales of grill products, shoarma, kebab and alike. |
|  | 59.210.180-Takeaway/ delivery | Provision of (hot) meals, which are not consumed on site but are collected or delivered |
| Full service restaurants | 59.210.235-Hotel-Restaurant | Hotel combined with ‘a la carte’ restaurant |
|  | 59.210.333-Lunchroom | Meal provider, with table service, particularly including breakfasts, lunches and desserts, opposed to restaurants mostly closed in the evening |
|  | 59.210.430-Café-Restaurant | Provision of both beverages and meals |
|  | 59.210.434-Restaurant | Provision of meals, beverages are provided only in conjunction with the food |
|  | 59.210.392-Pancakes | Restaurants specializing in pancakes |
| Supermarkets | 11.010.519-Supermarket | Stores with a wide and varied range of food products often supplemented by a narrow and shallow range of non-food products. Floor space is greater than 149m² |
| Greengrocers | 11.010.012-Vegetables/ fruit | Almost exclusively sales of potatoes, vegetables and fruit that have not been cultivated by the company itself |
| Shops selling animal products | 11.010.471-Butcher | Sales of meat and meat products |
|  | 11.010.399-Poulterer | Sales of game and poultry |
|  | 11.010.588-Fish | Sales of fish, crustaceans and molluscs |
| Bakeries | 11.010.111-Baker | Sales of bread and pastries, with possible lunchroom but that is not be the main activity |
|  | 11.010.112-Flans | Sales of pastries, with an emphasis on flans |
| Other shops | 11.010.132-Chocolate | Sales of Chocolate |
|  | 11.010.261-Cheese | Sales of Cheese |
|  | 11.010.310-Farm store | Farm sales of own products, primarily food products, supplemented by an assortment of purchased products |
|  | 11.010.378-Nuts | Sales of nuts, dates and dried fruits |
|  | 11.010.423-Reform | Biodynamic, ecological and macrobiotically grown products supplemented with dietary supplements, homeopathic remedies, herbs |
| Convenience stores | 11.010.309-Minisuper | See definition of supermarket, but store is up to 149m² |
|  | 11.010.350-Nightshop | Mini supermarket with (late) evening and night opening |
| Confectionary stores | 11.010.657-Sweets | Sales of sweets and confectionery, also sugars |
|  | 59.210.246-Ice cream parlor | Sales of ice cream |
| Excluded from the study | 11.010.137-Coffee/Tea | Store selling mainly coffee and tea, possibly supplemented by items intended for drinking coffee/tea |
|  | 11.010.424-Food Supplements | Sales of supplements intended to supplement or replace a normal diet. Also vitamins, sports supplements |
|  | 11.010.141-Delicacies | Store that sells specialty, usually more luxurious foods, and often convenience products |
|  | 11.010.477-Liquor | Sales of alcoholic and non-alcoholic beverages |
|  | 59.210.123-Bar | Provision of almost exclusively beverages (not meals) |
|  | 59.210.127-Coffee house | Provision of only non-alcoholic beverages (in the regulation Turkish or Moroccan beverages) |
|  | 59.210.334-Coffee bar | Provision of predominantly (hot, non-alcoholic) beverages prepared by a barista, limited offering of breakfasts, lunches and desserts. Predominantly ordering at the counter. |
|  | 59.210.950-Horeca other | Hospitality industry not further classifiable into one of the other categories |
|  | 11.010.123-Toko | Store selling mainly oriental foods |
