# Supplementary material for: Changes in retail food environments around schools over 12 years and associations with overweight and obesity among children and adolescents in Flanders, Belgium
Source: BMC Public Health. 2022 Aug 18;22:1570. doi: 10.1186/s12889-022-13970-8 (PMC9387020; doi:10.1186/s12889-022-13970-8)
Supplement: Supplementary file 2 — Additional file 2: Table S2. The average percentage of Flemish children with overweight stratified by age group and sex and school year. [file 12889_2022_13970_MOESM2_ESM.docx]

**Additional file 2: The average percentage of Flemish children with overweight stratified by age group and sex and school year**

*Table S2: The average percentage of Flemish children with overweight stratified by age group and sex and school year*

| **Age group** | **Sex** | **School year** | **N schools** | **% of children with overweight** | | |
| --- | --- | --- | --- | --- | --- | --- |
|  |  |  |  | **mean** | **lower 95%CI** | **upper 95%CI** |
| **<6years** | **boys** | 2010 - 2011 | 2190 | 7,89 | 7,64 | 8,15 |
|  |  | 2011 - 2012 | 2232 | 8,04 | 7,78 | 8,29 |
|  |  | 2012 - 2013 | 2244 | 7,79 | 7,53 | 8,05 |
|  |  | 2013 - 2014 | 2344 | 7,80 | 7,54 | 8,05 |
|  |  | 2014 - 2015 | 2370 | 7,98 | 7,73 | 8,24 |
|  |  | 2015 - 2016 | 2372 | 7,86 | 7,61 | 8,11 |
|  | **girls** | 2010 - 2011 | 2172 | 9,88 | 9,59 | 10,17 |
|  |  | 2011 - 2012 | 2198 | 10,30 | 10,02 | 10,59 |
|  |  | 2012 - 2013 | 2209 | 10,41 | 10,11 | 10,72 |
|  |  | 2013 - 2014 | 2304 | 10,30 | 10,01 | 10,59 |
|  |  | 2014 - 2015 | 2325 | 10,36 | 10,08 | 10,64 |
|  |  | 2015 - 2016 | 2332 | 10,52 | 10,23 | 10,81 |
| **6-12 years** | **boys** | 2010 - 2011 | 3102 | 12,29 | 12,02 | 12,56 |
|  |  | 2011 - 2012 | 3131 | 12,52 | 12,25 | 12,78 |
|  |  | 2012 - 2013 | 3188 | 12,46 | 12,18 | 12,74 |
|  |  | 2013 - 2014 | 3102 | 12,35 | 12,08 | 12,62 |
|  |  | 2014 - 2015 | 3128 | 12,32 | 12,05 | 12,59 |
|  |  | 2015 - 2016 | 3133 | 12,32 | 12,05 | 12,58 |
|  | **girls** | 2010 - 2011 | 3056 | 14,53 | 14,24 | 14,82 |
|  |  | 2011 - 2012 | 3088 | 14,77 | 14,48 | 15,07 |
|  |  | 2012 - 2013 | 3112 | 14,77 | 14,47 | 15,07 |
|  |  | 2013 - 2014 | 3013 | 14,77 | 14,49 | 15,06 |
|  |  | 2014 - 2015 | 3051 | 14,65 | 14,38 | 14,93 |
|  |  | 2015 - 2016 | 3052 | 14,90 | 14,62 | 15,19 |
| **13-14 years** | **boys** | 2010 - 2011 | 2180 | 13,05 | 12,56 | 13,55 |
|  |  | 2011 - 2012 | 2310 | 13,40 | 12,94 | 13,87 |
|  |  | 2012 - 2013 | 1666 | 13,41 | 12,86 | 13,97 |
|  |  | 2013 - 2014 | 929 | 14,24 | 13,60 | 14,88 |
|  |  | 2014 - 2015 | 924 | 14,61 | 13,96 | 15,25 |
|  |  | 2015 - 2016 | 918 | 14,33 | 13,69 | 14,97 |
|  | **girls** | 2010 - 2011 | 2047 | 13,99 | 13,45 | 14,53 |
|  |  | 2011 - 2012 | 2233 | 14,47 | 13,96 | 14,97 |
|  |  | 2012 - 2013 | 1581 | 14,43 | 13,81 | 15,05 |
|  |  | 2013 - 2014 | 829 | 16,12 | 15,41 | 16,83 |
|  |  | 2014 - 2015 | 826 | 16,23 | 15,50 | 16,96 |
|  |  | 2015 - 2016 | 814 | 16,71 | 15,99 | 17,44 |
| **14+ years** | **boys** | 2010 - 2011 | 668 | 13,95 | 13,08 | 14,82 |
|  |  | 2011 - 2012 | 637 | 15,70 | 14,70 | 16,70 |
|  |  | 2012 - 2013 | 555 | 14,62 | 13,61 | 15,62 |
|  |  | 2013 - 2014 | 548 | 15,13 | 14,18 | 16,08 |
|  |  | 2014 - 2015 | 524 | 15,56 | 14,65 | 16,47 |
|  |  | 2015 - 2016 | 502 | 14,89 | 13,90 | 15,88 |
|  | **girls** | 2010 - 2011 | 518 | 17,82 | 16,65 | 18,99 |
|  |  | 2011 - 2012 | 481 | 17,85 | 16,57 | 19,13 |
|  |  | 2012 - 2013 | 408 | 18,31 | 16,98 | 19,65 |
|  |  | 2013 - 2014 | 459 | 17,94 | 16,83 | 19,04 |
|  |  | 2014 - 2015 | 456 | 18,96 | 17,78 | 20,13 |
|  |  | 2015 - 2016 | 443 | 19,03 | 17,85 | 20,21 |
