# Supplementary material for: Changes in retail food environments around schools over 12 years and associations with overweight and obesity among children and adolescents in Flanders, Belgium
Source: BMC Public Health. 2022 Aug 18;22:1570. doi: 10.1186/s12889-022-13970-8 (PMC9387020; doi:10.1186/s12889-022-13970-8)
Supplement: Supplementary file 3 — Additional file 3: Table S3. Median (IQR) shortest distance (in meters) from primary/secondary schools to the closest convenience store, fast food/takeaway/delivery outlet and supermarket (years 2008 & 2020). [file 12889_2022_13970_MOESM3_ESM.docx]

**Additional file 3: Median shortest distance (in meters) and IQR r from primary and secondary schools to the nearest food retailer of a certain type.**

Table S3: Median (IQR) shortest distance (in meters) from primary/secondary schools to the closest convenience store, fast food/takeaway/delivery outlet and supermarket (years 2008 & 2020)

|  |  |  | *2008* | | | | *2020* | | | |
| --- | --- | --- | --- | --- | --- | --- | --- | --- | --- | --- |
| *School type* | *outlet type* | *N schools* | *median* | *P25* | *P75* | *max* | *median* | *P25* | *P75* | *max* |
| *primary schools* | *Convenience- or confectionary stores* | *3404* | 567.9 | 273.1 | 1617.2 | 9140.4 | 525.9 | 263.1 | 1464 | 7794.8 |
|  | *fast food/takeaway/delivery outlets* | *3404* | 386 | 213.6 | 774.1 | 7216.2 | 369.2 | 202 | 728.4 | 11008.1 |
|  | *supermarkets* | *3404* | 595.6 | 335.1 | 1316.3 | 10434 | 640.3 | 353.7 | 1432.4 | 7532.2 |
| *Secondary schools* | *Convenience- or confectionary stores* | *1195* | 429.1 | 233.6 | 798.1 | 5661.6 | 379.8 | 208.5 | 749.3 | 7771.6 |
|  | *fast food/takeaway/delivery outlets* | *1195* | 343.5 | 185.5 | 600.7 | 7866.8 | 325.3 | 172.7 | 574.7 | 7767.6 |
|  | *supermarkets* | *1195* | 518.6 | 336 | 845.8 | 6015.5 | 501.3 | 321.3 | 747.1 | 7890.4 |
