# Supplementary material for: Changes in retail food environments around schools over 12 years and associations with overweight and obesity among children and adolescents in Flanders, Belgium
Source: BMC Public Health. 2022 Aug 18;22:1570. doi: 10.1186/s12889-022-13970-8 (PMC9387020; doi:10.1186/s12889-022-13970-8)
Supplement: Supplementary file 4 — Additional file 4: Figure S1. Mean absolute density of fast food, takeaway and delivery outlets within 500m and 1000m road network distance from the entrance of primary schools in Flanders (year=2020) according to low, medium and high percentage of pupils with home language not being Dutch by level of urbanization of the municipality where the school is located. Figure S2. Mean absolute density (with 95% CI) of fast food, takeaway and delivery outlets within 500m and 1000m road network distance from the entrance of primary/secondary schools in Flanders (year=2020) according to low, medium and high percentage of pupils whose home language is not Dutch. Figure S3. Mean absolute density (with 95% CI) of convenience stores within 500m and 1000m road network distance from the entrance of primary/secondary schools in Flanders (year=2020) according to low, medium and high percentage of pupils whose home language is not Dutch. Figure S4. Mean absolute density of fast food, takeaway and delivery outlets within 500m and 1000m road network distance from the entrance of primary schools in Flanders (year=2020) according to low, medium and high percentage of pupils with a low educated mother by level of urbanization of the municipality where the school is located. [file 12889_2022_13970_MOESM4_ESM.docx]

**Additional file 4:**

**Mean absolute density of fast food, takeaway and delivery outlets, by level of urbanization and SES tercile based on students whose home language is not Dutch**


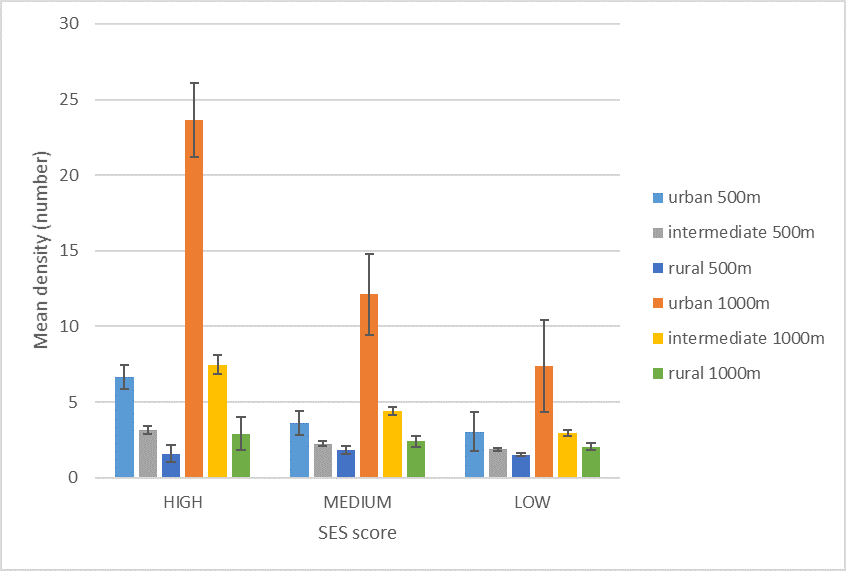


Figure S1: *Mean absolute density of fast food, takeaway and delivery outlets within 500m and 1000m road network distance from the entrance of primary schools in Flanders (year=2020) according to low, medium and high percentage of pupils with home language not being Dutch by level of urbanization of the municipality where the school is located.*

**Mean absolute density of fast-food, takeaway an delivery outlets by SES tercile based on the % of children whose home language is not Dutch, for primary and secondary schools separately**


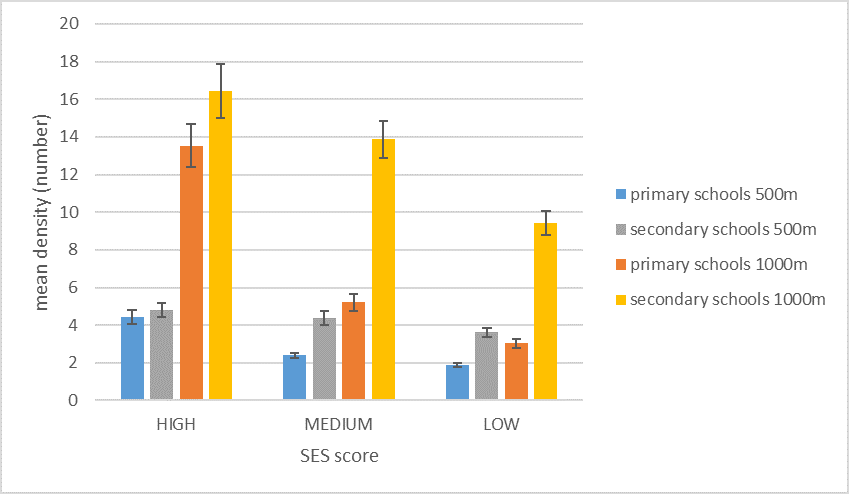


Figure S2: Mean absolute density (with 95% CI) of fast food, takeaway and delivery outlets within 500m and 1000m road network distance from the entrance of primary/secondary schools in Flanders (year=2020) according to low, medium and high percentage of pupils whose home language is not Dutch.

**Mean absolute density of convenience stores by SES tercile based on the % of children whose home language is not Dutch, for primary and secondary schools separately.**


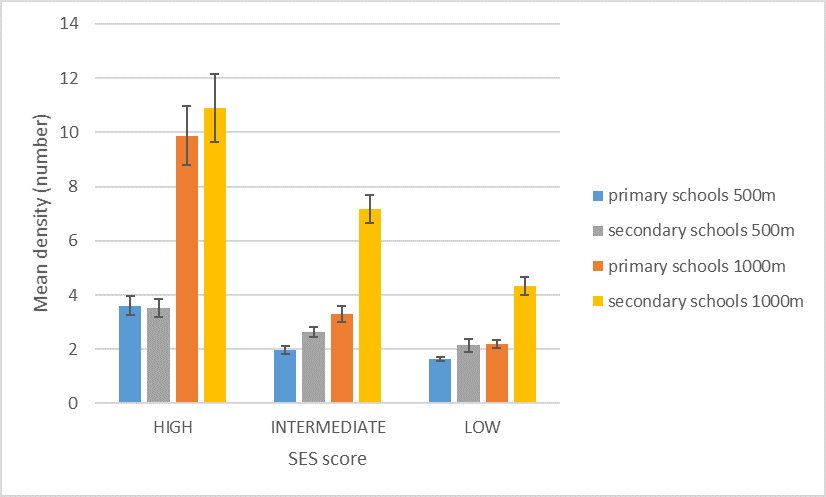


Figure S3: Mean absolute density (with 95% CI) of convenience stores within 500m and 1000m road network distance from the entrance of primary/secondary schools in Flanders (year=2020) according to low, medium and high percentage of pupils whose home language is not Dutch

**Mean absolute density of fast-food, takeaway an delivery outlets by level of urbanization and by SES tercile based on the % of children with a low educated mother.**

*
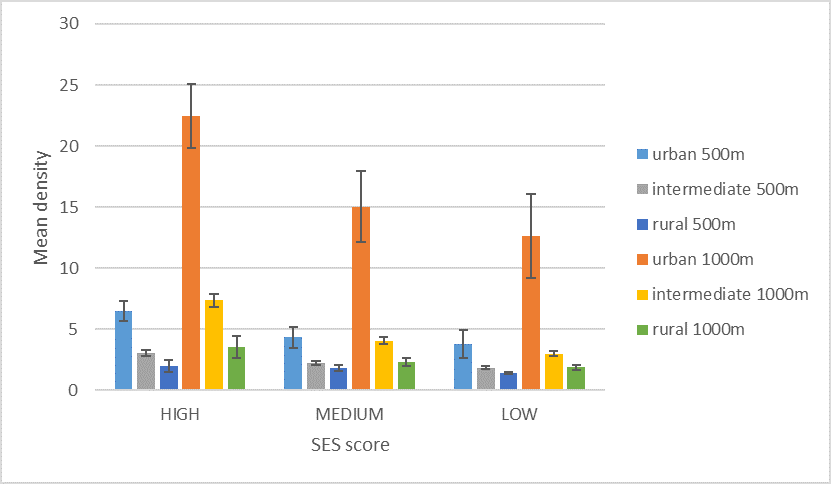
*

*Figure S4:* *Mean absolute density of fast food, takeaway and delivery outlets within 500m and 1000m road network distance from the entrance of primary schools in Flanders (year=2020) according to low, medium and high percentage of pupils with a low educated mother by level of urbanization of the municipality where the school is located*
