# Supplementary material for: Changes in retail food environments around schools over 12 years and associations with overweight and obesity among children and adolescents in Flanders, Belgium
Source: BMC Public Health. 2022 Aug 18;22:1570. doi: 10.1186/s12889-022-13970-8 (PMC9387020; doi:10.1186/s12889-022-13970-8)
Supplement: Supplementary file 5 — Additional file 5: Table S4. Associations between the density of convenience stores around schools and the percentage of the schools’ children with overweight, by age group and buffer (500m/1000m), adjusted for sex, level of urbanicity of municipality and % of pupils with a low educated mother. [file 12889_2022_13970_MOESM5_ESM.docx]

**Additional file 5: Associations between the density of convenience stores around schools and the percentage of the schools’ children with overweight, by age group, adjusted for sex, level of urbanicity of municipality and % of pupils with a low educated mother.**

Table S4: Associations between the density of convenience stores around schools and the percentage of the schools’ children with overweight, by age group and buffer (500m/1000m), adjusted for sex, level of urbanicity of municipality and % of pupils with a low educated mother.

| school year | Locatus year | outlet | buffer | Indicator weight status | Age group | Crude | | Adjusted | |
| --- | --- | --- | --- | --- | --- | --- | --- | --- | --- |
|  |  |  |  |  |  | Coefficient/SE | p | Coefficient/SE | p |
| 2010-2011 | 2008 | convenience | 500m | %overweight | Younger than 6 years | 0.214 / 0.029 | <0.001 | 0.123 / 0.028 | <0.001 |
| 2010-2011 | 2008 | convenience | 500m | %overweight | 6-12 years | 0.179 / 0.030 | <0.001 | 0.072 / 0.029 | 0.013 |
| 2010-2011 | 2008 | convenience | 500m | %overweight | 13-14 years | 0.171 / 0.051 | <0.001 | 0.139 / 0.051 | 0.007 |
| 2010-2011 | 2008 | convenience | 500m | %overweight | 15-18 years | 0.184 / 0.076 | 0.015 | 0.173 / 0.082 | 0.034 |
| 2010-2011 | 2008 | convenience | 1000m | %overweight | Younger than 6 years | 0.099 / 0.010 | <0.001 | 0.052 / 0.011 | <0.001 |
| 2010-2011 | 2008 | convenience | 1000m | %overweight | 6-12 years | 0.106 / 0.010 | <0.001 | 0.054 / 0.011 | <0.001 |
| 2010-2011 | 2008 | convenience | 1000m | %overweight | 13-14 years | 0.067 / 0.016 | <0.001 | 0.045 / 0.018 | 0.014 |
| 2010-2011 | 2008 | convenience | 1000m | %overweight | 15-18 years | 0.064 / 0.023 | 0.005 | 0.069 / 0.027 | 0.012 |
| 2015-16 | 2013 | convenience | 500m | %overweight | Younger than 6 years | 0.178 / 0.023 | <0.001 | 0.078 / 0.022 | <0.001 |
| 2015-16 | 2013 | convenience | 500m | %overweight | 6-12 years | 0.237 / 0.024 | <0.001 | 0.080 / 0.023 | <0.001 |
| 2015-16 | 2013 | convenience | 500m | %overweight | 13-14 years | 0.123 / 0.044 | 0.006 | 0.068 / 0.043 | 0.112 |
| 2015-16 | 2013 | convenience | 500m | %overweight | 15-18 years | -0.003 / 0.065 | 0.968 | -0.052 / 0.065 | 0.429 |
| 2015-16 | 2013 | convenience | 1000m | %overweight | Younger than 6 years | 0.090 / 0.008 | <0.001 | 0.043 / 0.009 | <0.001 |
| 2015-16 | 2013 | convenience | 1000m | %overweight | 6-12 years | 0.117 / 0.008 | <0.001 | 0.045 / 0.009 | <0.001 |
| 2015-16 | 2013 | convenience | 1000m | %overweight | 13-14 years | 0.089 /0.014 | <0.001 | 0.044 / 0.016 | 0.005 |
| 2015-16 | 2013 | convenience | 1000m | %overweight | 15-18 years | 0.013 / 0.021 | 0.531 | -0.021 / 0.023 | 0.377 |
| 2013-14 | 2013 | convenience | 500m | %overweight | Younger than 6 years | 0.184 / 0.024 | <0.001 | 0.067 / 0.024 | 0.004 |
| 2013-14 | 2013 | convenience | 500m | %overweight | 6-12 years | 0.247 / 0.024 | <0.001 | 0.123 / 0.023 | <0.001 |
| 2013-14 | 2013 | convenience | 500m | %overweight | 13-14 years | 0.056 / 0.044 | 0.205 | 0.035 / 0.043 | 0.421 |
| 2013-14 | 2013 | convenience | 500m | %overweight | 15-18 years | 0.064 / 0.062 | 0.301 | 0.043 / 0.063 | 0.496 |
| 2013-14 | 2013 | convenience | 1000m | %overweight | Younger than 6 years | 0.091 / 0.009 | <0.001 | 0.034 / 0.009 | <0.001 |
| 2013-14 | 2013 | convenience | 1000m | %overweight | 6-12 years | 0.120 / 0.008 | <0.001 | 0.060 / 0.009 | <0.001 |
| 2013-14 | 2013 | convenience | 1000m | %overweight | 13-14 years | 0.061 / 0.014 | <0.001 | 0.040 / 0.016 | 0.013 |
| 2013-14 | 2013 | convenience | 1000m | %overweight | 15-18 years | 0.054 / 0.020 | 0.009 | 0.046 / 0.022 | 0.038 |
| 2014-15 | 2013 | convenience | 500m | %overweight | Younger than 6 years | 0.294 / 0.026 | <0.001 | 0.111 / 0.031 | <0.001 |
| 2014-15 | 2013 | convenience | 500m | %overweight | 6-12 years | 0.335 / 0.026 | <0.001 | 0.149 / 0.031 | <0.001 |
| 2014-15 | 2013 | convenience | 500m | %overweight | 13-14 years | 0.200 / 0.050 | <0.001 | 0.192 / 0.060 | 0.001 |
| 2014-15 | 2013 | convenience | 500m | %overweight | 15-18 years | 0.117 / 0.070 | 0.098 | 0.013 / 0.084 | 0.873 |
| 2014-15 | 2013 | convenience | 1000m | %overweight | Younger than 6 years | 0.102 / 0.008 | <0.001 | 0.052 / 0.010 | <0.001 |
| 2014-15 | 2013 | convenience | 1000m | %overweight | 6-12 years | 0.113 / 0.008 | <0.001 | 0.059 / 0.009 | <0.001 |
| 2014-15 | 2013 | convenience | 1000m | %overweight | 13-14 years | 0.067 / 0.014 | <0.001 | 0.057 / 0.017 | <0.001 |
| 2014-15 | 2013 | convenience | 1000m | %overweight | 15-18 years | 0.027 / 0.021 | 0.189 | 0.006 / 0.024 | 0.807 |
